# A polymorphism of *EGFR *extracellular domain is associated with progression free-survival in metastatic colorectal cancer patients receiving cetuximab-based treatment

**DOI:** 10.1186/1471-2407-8-169

**Published:** 2008-06-10

**Authors:** Anthony Gonçalves, Séverine Esteyries, Brynn Taylor-Smedra, Arnaud Lagarde, Mounay Ayadi, Geneviève Monges, François Bertucci, Benjamin Esterni, Jean-Robert Delpero, Olivier Turrini, Bernard Lelong, Patrice Viens, Jean-Paul Borg, Daniel Birnbaum, Sylviane Olschwang, Frédéric Viret

**Affiliations:** 1 Department of Medical Oncology, Institut Paoli-Calmettes, Marseille, France; 2 Department of Molecular Pharmacology, Institut Paoli-Calmettes, Marseille, France; 3 Department of Molecular Oncology, Institut Paoli-Calmettes, Marseille, France; 4 Department of BioPathology, Institut Paoli-Calmettes, Marseille, France; 5 Department of Surgical Oncology, Institut Paoli-Calmettes, Marseille, France; 6 Université de la Méditerranée, UFR de Médecine, Marseille, France; 7 INSERM U891 ; Centre de Recherche en Cancérologie de Marseille, Marseille, France

## Abstract

**Background:**

Cetuximab, a monoclonal antibody targeting Epidermal Growth Factor Receptor (EGFR), is currently used in metastatic colorectal cancer (mCRC), but predictive factors for therapeutic response are lacking. Mutational status of *KRAS *and *EGFR*, and *EGFR *copy number are potential determinants of cetuximab activity.

**Methods:**

We analyzed tumor tissues from 32 EGFR-positive mCRC patients receiving cetuximab/irinotecan combination and evaluable for treatment response. *EGFR *copy number was quantified by fluorescence in situ hybridization (FISH). *KRAS *exon 1 and *EGFR *exons coding for extracellular regions were sequenced.

**Results:**

Nine patients experienced an objective response (partial response) and 23 were considered as nonresponders (12 with stable disease and 11 with progressive disease). There was no *EGFR *amplification found, but high polysomy was noted in 2 patients, both of which were cetuximab responders. No *EGFR *mutations were found but a variant of exon 13 (R521K) was observed in 12 patients, 11 of which achieved objective response or stable disease. Progression-free and overall survivals were significantly better in patients with this *EGFR *exon 13 variant. *KRAS *mutations were found in 14 cases. While there was a trend for an increased *KRAS *mutation frequency in nonresponder patients (12 mutations out of 23, 52%) as compared to responder patients (2 out of 9, 22%), authentic tumor response or long-term disease stabilization was found in *KRAS *mutated patients.

**Conclusion:**

This preliminary study suggests that: an increase in *EGFR *copy number may be associated with cetuximab response but is a rare event in CRC, *KRAS *mutations are associated with low response rate but do not preclude any cetuximab-based combination efficacy and *EGFR *exon 13 variant (R521K) may predict for cetuximab benefit.

## Background

Epidermal growth factor receptor (EGFR), which participates in signaling pathways that are deregulated in cancer cells, is a promising target in epithelial cancer, notably colorectal cancer [1]. Cetuximab (Erbitux^®^), a monoclonal antibody targeting EGFR, is currently used in EGFR-expressing metastatic colorectal cancer (mCRC) in combination with cytotoxic chemotherapy (irinotecan), after failure of a previous irinotecan-based regimen. In this setting, cetuximab produces objective response in about 25% of patients, with nearly 30% of patients achieving disease stabilization [2], resulting in a median progression-free survival of 4 months and a median overall survival of 6 to 9 months. Recently, another EGFR-targeted monoclonal antibody, panitumumab (vectibix^®^) was FDA-approved in mCRC as single agent, after failing chemotherapy drugs fluoropyrimidine, oxaliplatin and irinotecan[3]. Panitumumab induces a response rate of 10%, similar to that achieved with single-agent cetuximab in a similar patient population [4,5], and demonstrates a modest but significant increase in median progression-free survival against best supportive care.

Clearly, clinical benefit with EGFR-targeting antibodies seems to be restricted to a particular subgroup of mCRC patients. However, no validated predictive factor is currently available to improve the rational administration of these therapies in this patient population. Such factors are critically needed, especially if we consider the high cost of these new therapeutics[6] and their expected future integration in regimens administered in earlier clinical stages, including first-line treatment of mCRC and adjuvant chemotherapy in stage III localized disease.

Somatic mutations of EGFR tyrosine kinase domain are associated with exquisite sensitivity to EGFR-tyrosine kinase inhibitors erlotinib and gefitinib in non-small cell lung cancer (NSCLC) [7-9], but such mutations are rare or absent in CRC [5,10]. EGFR protein expression, as evaluated by immunohistochemistry, does not correlate with response [2,4,11] and only specific treatment-induced skin rash seems associated with tumor response and progression-free survival [2].

Recent retrospective data have suggested that *EGFR *amplification or *KRAS *exon 1 somatic mutations may allow a better selection of patients who are candidates for EGFR targeting [12,13]. In a first study [12], an increase in *EGFR *copy number identified by FISH (fluorescence in situ hybridization) was found in all but one responding patients, while four recent studies identified no or few cetuximab responders in *KRAS *mutated patients [13-17].

In the present retrospective study of 32 patients with EGFR-positive mCRC treated with cetuximab-based combination, we have analyzed *EGFR *copy number by FISH and sequenced the extracellular domains (ECD) of *EGFR*, as well as *KRAS *exon 1, and correlated these data with clinical outcome.

## Methods

### Patients and treatment

We retrospectively assessed 32 patients with EGFR-positive mCRC treated with cetuximab-irinotecan combination at the Institut PAOLI-CALMETTES, Marseille, France between March 2004 and July 2005 who were evaluable for tumor response and had available pre-treatment frozen and/or formalin-fixed and paraffin-embedded tumor tissues (from primary and/or metastatic tumor tissue). EGFR positivity was defined by at least 1% malignant cells demonstrating EGFR immunostaining (antibody from Zymed Laboratories, Inc., San Francisco, 1/20, digested with pepsin). In 26 patients, cetuximab was used according to official registration in irinotecan-resistant patients and administered as a loading dose of 400 mg/m2 intravenously, followed by 250 mg/m2 once a week until progression, in combination with irinotecan 180 mg/m^2 ^every other week. In addition, 6 patients received cetuximab/irinotecan in specific clinical trials evaluating:

- a dose escalation of cetuximab in irinotecan-resistant patients (n = 3)

- cetuximab in combination with Folfiri as first-line treatment (n = 2) or as second-line treatment after failure of first-line fluoropyrimidine/oxaliplatin (n = 1).

Tumor evaluation was performed with appropriate methods within four weeks of treatment initiation and repeated during treatment every 2 months for the first 6 months and then every 3 months until disease progression. WHO criteria of response were used [18]. Briefly, the sum of products of target lesions was calculated and response was determined as follows: complete response (CR), disappearance of all target lesions without any residual lesion; partial response (PR), 50% or more decrease in target lesions; progressive disease (PD), 25% or more increase in the size of measurable lesions or appearance of new lesions; stable disease (SD), neither PR or PD criteria are met. This study was approved by an institutional review board and patient consent for analysis of stored biological samples, in relation with clinical data including imagery data, was verified for all patients included.

### EGFR copy number by FISH

Formalin-fixed paraffin-embedded (FFPE) tissue sections (5 μm) were placed in pretreatment solution for 60 min at 80°C, and digested with pepsin solution for 15 min at 37°C. Dual-color, dual-target FISH assays were done with the SPEC EGFR/CEN7 Dual Color Probe Kit (ZytoVision, Bremerhaven, Germany). Tissue sections, covered with 10-μL probe solution, were incubated at 75°C for 10 min to co-denature EGFR and CEN7 (chromosome seven α-centromeric) probes and allowed to hybridize overnight at 37°C. Codenaturation and hybridization were done sequentially in a microprocessor-controlled system (Hybridizer, DakoCytomation, Glostrup, Denmark). Posthybridization stringency wash was done in a water bath at 37°C for 5 min. After washing 4 times and drying at room temperature for 15 min, tissue sections were covered with 4'-6-diamidino-2-phenylindole (DAPI/Antifade Solution, ZytoVision, Bremerhaven, Germany) for chromatin counterstaining before microscopy. Sample material was evaluated by fluorescence microscopy (Leica. DM RXA). Filter sets for the following wavelength ranges were required: EGFR (ZyGreen), excitation at 503 nm and emission at 528 nm, similar to FITC; chromosome 7 (ZyOrange), excitation at 547 nm and emission at 572 nm, similar to Rhodamine.

Two independent observers (SE and AL) scored at least 100 non-overlapping interphase nuclei for the number of copies of EGFR and CEN7 by use of predefined scoring guidelines. The negative controls consisted of a healthy colorectal mucosa adjacent to malignant disease; the control for amplified EGFR was an amplified colonic adenocarcinoma. FISH patterns were defined as described in [19] : Briefly, the samples were grouped as follows: normal disomy, two gene copies in more than 90% of cells; trisomy, three gene copies in more than 10% of cells and ratio gene/chromosomes ≤ 2 ; low polysomy, at least four gene copies in more than 10% but fewer than 40% of cells and ratio gene/chromosomes ≤ 2; high polysomy, at least four gene copies in more than 40% cells and ratio gene/chromosomes ≤ 2; and gene amplification, ratio gene/chromosome more than two or 15 gene copies in at least 10% of cells. Trisomy and low polysomy were not considered as increases in *EGFR *copy number. Tumors showing high polysomy and/or gene amplification were considered to be FISH positive and as significant increases in *EGFR *copy number.

### DNA extraction and mutation analyses

DNA was extracted from frozen (n = 20) or FFPE colorectal cancer samples (n = 12) with the QIAamp DNA minikit (Qiagen). For FFPE tissues, samples were obtained by pinching tissue fragments within a tumor zone under microscopic control. Exon 1 of KRAS, the site of the most frequent activating mutations in codons 12 and 13, and exons 6 to 14 of EGFR, corresponding to the transmembrane and extracellular domains, were sequenced after PCR amplification of each exon using the BigDye terminator kit v1.1 (Applied Biosystems) and the PhredPhrapConsed package. Genotypes were assessed with the GeneMapper software (Applied Biosystems).

### Statistical analyses

Fischer's exact test was used to calculate p-values for the association between genetic parameters and response to cetuximab. Progression-free survival (PFS) was calculated from the date of cetuximab initiation to the date of disease progression, date of death if it occurred before progression, or date of last news. Overall survival (OS) was calculated from the date of cetuximab initiation to the date of death or the date of last news. Survivals were estimated with the Kaplan-Meier method. Survival curves were compared using the log-rank test. Analysis was carried out using the R software. The level of significance was set at p = 0.05.

## Results

### Patient population

Patient characteristics are summarized in table 1. Cetuximab was administered after failing 2 or more regimens in 22 patients. Twenty-four patients had been previously exposed to fluoropyrimidines, irinotecan and oxaliplatin. Median follow-up was 19.1 months. An objective response was observed in 9 patients (RR = 28%; CI95%, 15–45%), and 12 patients experienced stable disease, which lasted 6 months or more for 2 patients. Eleven patients progressed at the first evaluation. Median progression-free and overall survivals were 4.1 months (CI95%, 3.6–6.3) and 17.2 months (CI95%, 13.8-NR), respectively.

**Table 1:** Patient population

	Patients	
Characteristics	number	percentage

All patients	16	
Sex		
Male	16	50%
Female	16	50%
Age, years		
Median		58
Range		36–78
Tumor site		
Colon	21	65%
Rectum	11	35%
Previous adjuvant chemotherapy		
Yes	10	31%
No	22	69%
Line of cetuximab use		
Median		3
Range		1–5
Response status		
CR	0	
PR	9	28.1%
SD	12	37.5%
PD	11	34.4%

CR = Complete response, PR = Partial response, SD = Stable disease and PD = Progressive disease were defined as described in the methods section.

### EGFR copy numer analysis

**Figure 1:**
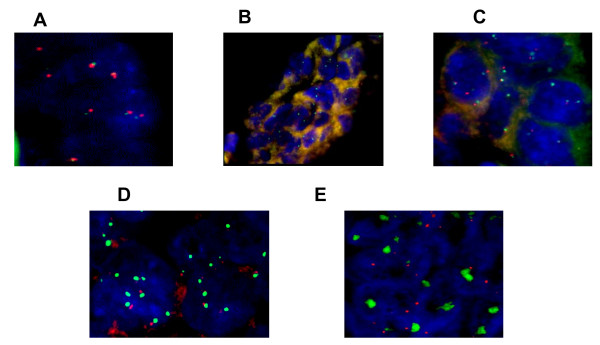
**Dual color FISH assays for probes of EGFR (green) and chromosome seven (CEP7, red)**. (A) Balanced disomy in healthy colorectal mucosa. (B) Balanced disomy in tumor of patient 15. (C) Balanced low polysomy in tumor of patient 4. (D) High polysomy in tumor of patient 1. (E) Amplification in a control tumor.

No authentic regional amplification was observed. An increased *EGFR *copy number was noted in 12 patients (Table 2), but was considered as significant in only 2 patients, corresponding to high polysomy (patients 1 and 3, Figure 1). Both patients responded to cetuximab.

**Table 2:** Molecular Alterations in tumors of metastatic colorectal cancer patients

Pt number	Sex	Age	Previous adjuvant CT	Type of adjuvant CT	Previous regimen for metastatic disease	Tumor Best response	Time to progression (weeks)	*KRAS *exon 1	*EGFR *exon13	*EGFR copy Number (FISH)*
***Responders***										
1	F	70	0		-	PR	10	Wild-type	R521K	high polysomy
2	M	62	0		Folfirinox	PR	94*	Wild-type		trisomy
3	F	56	0		Folfiri, Folfox	PR	9	Wild-type		high polysomy
4	M	37	0		Folfiri, Folfox, xelox	PR	44	Wild-type		low polysomy
5	F	44	0		Folfiri, Folfiri	PR	33	Wild-type	R521K	disomy
6	F	54	1	LV5FU	Folfiri	PR	67	Gly13Asp		disomy
7	F	52	1	Fufol	Folfirinox, Folfiri, LV5FU-mitomycine	PR	42	Gly12Asp	R521K	disomy
8	M	56	0		Foflfox	PR	25	Wild-type		disomy
9	M	66	1	Fufol	Folfiri, Folfox	PR	24*	Wild-type	R521K	trisomy
										
***Non-responders***										
10	F	68	0		Folfiri, Xeloda, Xelox	SD	17	Wild-type	R521K	trisomy
11	F	74	1	LV5FU	Folfiri	SD	10	Gly12Val		disomy
12	M	61	0		Folfirinox	SD	18	Gly13Asp	R521K	disomy
13	F	71	0		Xelox Avastin-Xeliri	SD	15	Gly12Asp		disomy
14	M	59	0		LV5FU, Folfiri, Folfox	SD	24	Gly12Cys	R521K	trisomy
15	M	71	0		Folfox, Irinotecan	SD	14	Wild-type		disomy
16	F	60	1	Fufol	Folfiri, Folfox, Cape, LV5FU	SD	17	Wild-type		trisomy
17	M	65	0		Folfirinox, Folfiri	SD	18	Wild-type		low polysomy
18	M	66	0		Folfiri	SD	17	Wild-type	R521K	disomy
19	F	45	0		Folfox, Xelox	SD	17	Wild-type	R521K	NE
20	F	42	0		Folfirinox	SD	21	Gly12Asp	R521K	trisomy
21	M	62	1	Folfox	Xeliri	SD	36	Gly13Asp	R521K	disomy
22	M	58	1	Folfiri	Xelox, Xeliri	PD	NA	Gly13Asp		disomy
23	M	75	1	Xelox	Xeliri	PD	NA	Wild-type		trisomy
24	M	81	0		Xelox, Folfiri	PD	NA	Gly12Val		disomy
25	F	55	0		Xelox Avastin-Xeliri	PD	NA	Gly12Asp		disomy
26	M	60	1	LV5FU	Folfiri, Folfiri, Folfox	PD	NA	Wild-type		NE
27	M	59	0		Folfiri, Folfiri/Folfox, Folfox	PD	NA	Gly12Asp		NE
28	F	58	1	LV5FU	Folfiri, Xelox	PD	NA	Gly12Asp		disomy
29	M	51	0		Folfox, Folfiri	PD	NA	Wild-type		disomy
30	F	66	0		Folfiri, Xelox	PD	NA	Wild-type	R521K	trisomy
31	F	56	0		-	PD	NA	Wild-type		disomy
32	F	78	0		Folfox, Folfiri, Cape	PD	NA	Gly12Val		disomy

F = female, M = Male, Folfox = oxaliplatin, fluorouracil, and folinic acid; Xelox = capecitabine, oxaliplatin; Folfiri = irinotecan, fluorouracil, and folinic acid; Xeliri = Capecitabine, irinotecan; Folfirinox = oxaliplatin, irinotecan, fluorouracil, and folinic acid; LV5FU = infusional FU and folinic acid; Fufol = bolus fluorouracil and folinic acid; PR = partial response. SD = stable disease. PD = progressive disease. R521K = point substitution G→A on exon 13 *EGFR *resulting in the amino acid substitution Arg to Lys in position 521. NA = not applicable. NE = not evaluable.

### EGFR sequencing

**Figure 2:**
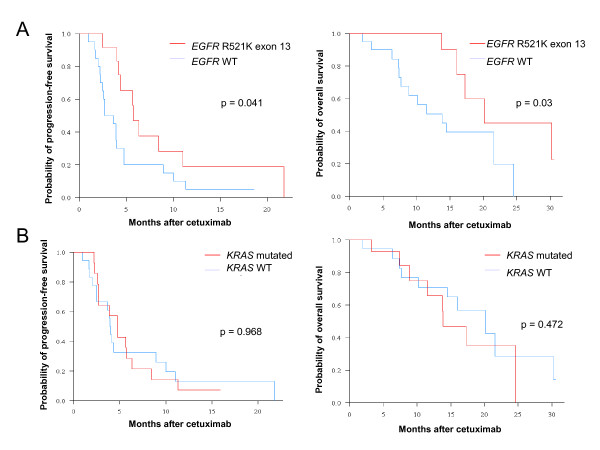
**Survivals according to *EGFR *and *KRAS *genotypes**. A/ Progression-free survival (PFS) (Left) and overall survival (OS) (Right) curves of patients with *EGFR *R521K variant and wild-type. B/ PFS (Left) and OS (Right) curves of patients with a *KRAS*-mutated and nonmutated tumor.

We sequenced *EGFR *exons 6 to 14 coding for the transmembrane and extracellular domains. No mutation was found, but a heterozygous (9 patients) or homozygous (3 patients) point substitution G→A on exon 13, resulting in the amino acid substitution of arginine by lysine in position 521 (R521K), was detected in 12 patients. This variant was observed in 11 of 21 patients achieving objective response (4 patients) or stable disease (7 patients) and in 1 of 11 patients with rapidly progressive disease (defined as progression at time of the first evaluation) (p = 0.02, Fischer's exact test) (Table 2). As shown in figure 2A, median PFS and OS were significantly better in patients with the R521K variant than in wild-type patients (5.7 [CI95%, 4.3-NR] vs. 3.2 [CI95%, 2.5–4.7] months; p = 0.041, log-rank test for PFS; 20.1 [CI95%, 16-NR] vs. 13.8 [CI95%, 7.7-NR] months; p = 0.03 log-rank for OS).

### KRAS mutations

Sequencing of *KRAS *exon 1 was performed and correlated with clinical outcome (Table 2). Fourteen patients displayed *KRAS *exon 1 mutations (codon 12 for 10 patients, codon 13 for 4 patients). Of the 32 genotyped tumors, 19 were metastases and 13 were primary tumors. In six patients, paired primary and metastatic tumors were available; no discordance was observed between the genotypes. In 9 responding patients, 2 had a *KRAS *mutation (22%), whereas 12 of 23 (52%) nonresponding patients had a *KRAS *mutation. This difference was not statistically significant (p = 0.234, Fischer's exact test). As shown in figure 2B, PFS was similar in *KRAS*-nonmutated and *KRAS*-mutated patients: median PFS was 3.9 months (CI 95%, 2.5–11) and 4.7 months (CI 95%, 2.7–11.3) (p = 0.968, log-rank test) in *KRAS*-nonmutated and *KRAS*-mutated patients, respectively. Overall survival was better in *KRAS*-nonmutated (20.8 months [CI 95%, 14.5-NR]) than in *KRAS*-mutated patients (13.8 months [CI 95%, 11.5-NR]), however this difference did not reach statistical significance (p = 0.472, log-rank test).

### Clinical features of KRAS-mutated benefiting from cetuximab based-treatment

**Figure 3:**
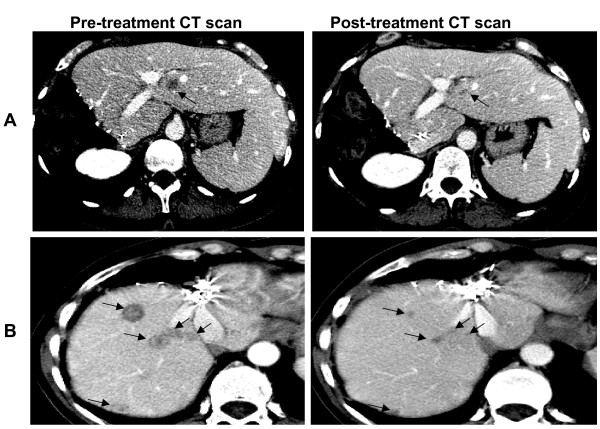
**Objective partial responses to cetuximab-based treatment in patients with *KRAS- *mutated tumors**. (A) Pre- and post-cetuximab CT scan showing partial tumor response allowing surgical resection to be performed in patient 6. (B) Pre- and post-cetuximab CT scan demonstrating a major tumor response to cetuximab-based treatment in patient 7.

To better examine the clinical relevance of response or long-lasting stable disease obtained by cetuximab-irinotecan combination in 3 patients with KRAS-mutated tumors, their medical records were reviewed. The first patient (Pt 6) was a 53-year-old woman presenting liver metastases from a primary pT2N2 colon cancer resected in May 2003. She had received adjuvant LV-5FU (infusional 5FU/folinic acid) chemotherapy between May 2002 and October 2003. A first hepatic relapse occurred in January 2004 and was treated with two courses of Folfox (5FU, folinic acid, oxaliplatin) followed by Folfiri (5FU, folinic acid, irinotecan) due to allergic reaction after oxaliplatin infusion. The patient achieved a partial response and a right hepatectomy for metastasis removal was performed on April 2004, followed by six cycles of Folfiri in August 2004. She had a second hepatic relapse in September 2004 and in October 2004 was subjected to radiofrequency ablation which failed, resulting in the appearance of an additional hepatic lesion. The patient started cetuximab in association with irinotecan in January 2005 and achieved an authentic partial response allowing surgical removal in April 2005 (Figure 3A). Surgery was pathologically complete and the patient was alive in complete remission at time of last news in May 2006.

The second responding patient with a *KRAS *mutation (Pt 7) was a 52-year-old woman who had been treated in June 1999 for a primary adenocarcinoma of the rectum classified pT4N2. The patient received post-operative radio-chemotherapy with Fufol (Bolus 5FU/folinic acid) on week 1 and 5. In February 2000, she had liver metastases that were completely surgically removed. In December 2000, she had a second hepatic relapse and received an association of 5FU/irinotecan/oxaliplatin for 2 months allowing her to be a candidate for a second complete surgical removal which was performed in April 2001. On January 2003, she experienced a third relapse involving retroperitoneal lymph nodes which was treated with Folfiri until May 2003, when a partial response was achieved. Maintenance with capecitabine was initiated and continued until October 2003 when disease progressed on both lymph nodes and the liver. A palliative treatment was started combining 5FU and mitomycin C with no response. The patient started cetuximab with irinotecan in March 2004, when the compound became available in France. She experienced a partial response until January 2005 (Figure 3B), when she developed bone metastases with spinal compression. She died in February 2005.

Finally, a third patient (pt 21) with a *KRAS *mutation achieved long-lasting stable disease (8 months), under cetuximab-irinotecan treatment. This 62-year old man had been treated in 2004 for a pT3N2 colon cancer by surgery followed by adjuvant Folfox. In December 2004, while he was receiving Folfox, he developed liver metastases which were surgically removed. Post-operative treatment was initiated in March 2005 with an association of capecitabine/irinotecan, but lung metastases occurred under treatment. A combination of cetuximab and irinotecan was started in June 2005 and continued with stable disease until February 2006 when peritoneal and lung progression occurred. The patient died in August 2006.

## Discussion

In this retrospective and preliminary study including 32 mCRC patients receiving cetuximab-based treatment, we have analyzed molecular factors that have been suggested to regulate activity of EGFR-targeted approaches.

### EGFR copy number

Using a FISH-based assay, we evaluated *EGFR *copy number and did not observe any *EGFR *amplification (ratio *EGFR *to Control probes >2 or ≥ 15 gene copies in ≥ 10% of cells), although trisomy or polysomy was observed in 12 patients. Only 2 patients displayed an increase in *EGFR *copy number that reached the definition of high polysomy (≥ four gene copies in ≥ 40% cells). However, these 2 patients were objective responders to cetuximab. In previous studies, an increase in *EGFR *gene copy number has been observed in 0.6 to 31% of CRC. These variations may be due in part to the techniques used to evaluate amplification, which include FISH, CISH or real-time quantitative PCR. Moreover, amplifications limited to the *EGFR *locus must be distinguished from extracopies of the entire chromosome 7 which contains the *EGFR *gene (polysomy). In a study involving 31 patients[12], Moroni et al observed an increase in *EGFR *copy number in 8 of the 9 cetuximab-responders but only in 1 of the 20 nonresponder patients who were assessable by FISH, suggesting that this increase may represent a strong positive predictive factor for response to this compound. Of note, an authentic amplification was found in 7 of these 9 patients with increased *EGFR *copy number. However, the same group recently reported no actual *EGFR *amplification but only polysomy in 58 FISH-analyzed tumors from mCRC patients receiving panitumumab. These authors have concluded that the previously observed amplification frequency could have been overestimated by scoring as amplified some tumors with only very limited foci of amplification. Nevertheless, this study still suggested a significant association between *EGFR *copy number and response and survival [20]. Other groups have reported a lower rate of copy gain, corresponding most often to polysomy [13,21-23]. In one of these studies which is consistent with our own results, only 3 patients out of 30 displayed an increase in *EGFR *copies as evaluated by CISH [13]; these 3 patients responded to cetuximab. Thus, a significant increase in *EGFR *copy number may be associated with a high probability of response, even though this molecular alteration may be relatively rare in mCRC. Furthermore, regarding the significant number of responding patients without any significant increase in *EGFR *copy number in both Lievre et al's study (8 out of 11 responders) and ours (7 out of 9 responders), we do not believe that this parameter should be considered as a prerequisite for cetuximab activity.

### KRAS mutations

Recent retrospective data from several independent studies have shown a very negative association between *KRAS *exon 1 mutations and cetuximab response in mCRC patients. A first group found no *KRAS *mutations in tumors from 11 cetuximab-responding patients, whereas 13 of the 19 nonresponders (p = 0.0003) had a *KRAS*-mutated tumor, leading the authors to suggest that *KRAS *mutational status could serve to exclude cetuximab use in mCRC[13]. A second recently published study involving 59 refractory mCRC patients receiving cetuximab-based treatment confirmed these data by reporting no mutations in all 12 responding patients, with a significantly worse time-to-progression in patients with a *KRAS *mutation[14]. Additionally, a third report has shown that mutations affecting either *KRAS *or *BRAF *are predictive and prognostic indicators in mCRC patients, and are inversely correlated with response to anti-EGFR monoclonal antibodies [16]. Another study evaluating *KRAS/BRAF *mutation status in 80 patients receiving cetuximab as single-agent found only 3 *KRAS *mutated tumors out of 27 patients who experienced a clinical benefit, but 27 out of 53 nonresponding patients [15]. Finally, recent data from 27 mCRC patients observed only one responder out of 10 mutated tumors compared to 9 out of 17 non mutated tumors[17]. We found a similar trend in our study, although our observations did not reach statistical significance. Moreover, pooling all published studies evaluating this putative association further suggests that *KRAS *mutations strongly negatively affect the probability of objective response to cetuximab treatment. In these studies, the response rate in *KRAS *mutated tumors was 9 out of 115 (7.8%, CI95%: 3.6–14.3%) versus 81 out of 192 (42.2%, CI95%: 35.1–49.5%) in wild type tumors (p = 3.5 10^-10^, chi-2 test). However, we did not find any statistically significant difference in OS or PFS in patients with KRAS-mutated and wild-type tumors. Although this lack of difference may be reasonably attributed to the limited sample size, data presented in our study also demonstrated that authentic and clinically relevant tumor responses and/or long-term stabilization may be achieved with cetuximab-based treatment in patients with *KRAS*-mutated tumors. Accordingly, we believe that it could be premature to absolutely exclude cetuximab use in these patients.

### A variant of EGFR extracellular region

Activating mutations of the intracellular kinase domain of *EGFR *have been associated with human malignancies and responsiveness to small molecule EGFR tyrosine kinase inhibitors[5,10,12]. These mutations are rare or absent in mCRC, and are thus unlikely to explain the reported antitumor activity of cetuximab in this population. Nevertheless, little is known about the extracellular region of EGFR which represents the binding site of cetuximab. We sequenced this entire domain and did not find any mutations. However, we observed in 12 patients (37%) a G→A substitution in exon 13, which encodes a part of the extracellular region of the receptor. The resulting amino acid substitution Arg to Lys is located at the boundary between EGFR domain III, which represents the direct interaction site with cetuximab, and domain IV [24]. In our study, this variation was observed in 11 patients achieving at least a stable disease as their best response, but only in 1 patient with progressive disease at its first evaluation. Moreover, PFS and OS after cetuximab treatment were significantly better in the subset of patients displaying this variant.

This substitution, considered as a polymorphism (rs11543848 in SNPdb, heterozygosity of 0.41), may be relatively conservative, as both Arg and Lys are positively charged amino acids with similar side chains. It is also found in DNA from normal human lymphocytes [25] obtained from individuals without malignant diseases with a frequency of about 20% (homozygous variant) to 50% (heterozygous variant) in the general population [26].

Furthermore, *EGFR *exon 13 R521K variant has been already described in other EGFR expressing tumors, such as gliomas and lung cancer [27]. This *EGFR *polymorphism, previously described as codon 497 (R497K) according to an older nomenclature, has been negatively associated with pelvic recurrence in patients with rectal cancer treated with chemoradiation [28]. Recent data have shown that it correlates with a decrease in EGFR phosphorylation, decreased invasion, lower nodal involvement, reduced subsequent metastasis, and longer disease-free and overall survival in stage II/III colorectal carcinoma patients who have received curative surgery[26]. In addition, R521K was associated with oxaliplatin/FU efficacy in metastatic patients. Interestingly, the resulting amino acid substitution (Arg to Lys) was shown to significantly reduce TGFα binding and ligand-induced EGFR signaling [29]. Thus, it is tempting to speculate that *EGFR *variant may alter binding of its specific ligands leading to a particular phenotype of EGFR signaling. This is particularly interesting in light of very recent evidences generated from a microarray study showing that expression of EGFR ligands epiregulin and amphiregulin may predict cetuximab benefit [15]. An attenuated EGFR-mediated signaling, as putatively supposed with R521K polymorphism, could be even more sensitive to targeted receptor inhibition. Alternatively, R521K variant could also affect drug binding and/or effects. However, such preliminary hypotheses remain to be proven: we have initiated specific functional studies evaluating the correlation between cetuximab sensitivity and the *EGFR *exon 13 genotype in various CRC cell lines and in cellular models expressing wild type *EGFR *or the *EGFR *R521K variant. Importantly, EGFR genotyping on a larger cohort of cetuximab-treated patients, the accrual of which is currently ongoing, will be essential to confirm our findings.

## Conclusion

In conclusion, genetic factors affecting cetuximab response are likely to be multiple. *EGFR *copy number as well as variations in amino acid composition of the extracellular region may favorably impact cetuximab activity, whereas *KRAS *mutations negatively alter the probability of response, without totally abolishing it. However, these data from a small-sized patient population are still preliminary. Thus, validation of these results on larger cohorts and prospective studies are imperatively needed.

## Abbreviations

ECD: extracellular domain; EGFR: epidermal growth factor receptor; FISH: fluorescence in situ hybridization; mCRC: metastatic colorectal cancer.

## Competing interests

The authors declare that they have no competing interests.

## Authors' contributions

AG conceived of the study and its design and was in charge of its coordination. He participated in data analysis and performed data interpretation. He drafted the manuscript. SE carried out the FISH assay and helped to draft the manuscript. AL carried out the genetic analysis and participated in data analysis and interpretation. BT–S participated in the data analysis and interpretation and helped to draft the manuscript. MA participated in the data analysis. GM participated in the data analysis and interpretation. FB participated in the data interpretation and helped to draft the manuscript. BE performed the statistical analysis. J–RD, OT and BL participated in patient treatment and data acquisition. PV helped to conceive and coordinate the study. J–PB helped to draft the manuscript. DB participated in designing, coordinating the study, and in data interpretation. He helped to draft the manuscript. SO carried out the genetic analysis, participated in data interpretation and helped to draft the manuscript. FV was in charge of patient treatment, participated in study design, coordination, and data interpretation and helped to draft manuscript. All authors read and approved the final manuscript.

## Pre-publication history

The pre-publication history for this paper can be accessed here:



## References

[B1] Yarden Y, Sliwkowski MX (2001). Untangling the ErbB signalling network. Nat Rev Mol Cell Biol.

[B2] Cunningham D, Humblet Y, Siena S, Khayat D, Bleiberg H, Santoro A, Bets D, Mueser M, Harstrick A, Verslype C, Chau I, Van Cutsem E (2004). Cetuximab monotherapy and cetuximab plus irinotecan in irinotecan-refractory metastatic colorectal cancer. N Engl J Med.

[B3] Van Cutsem E, Peeters M, Siena S, Humblet Y, Hendlisz A, Neyns B, Canon JL, Van Laethem JL, Maurel J, Richardson G, Wolf M, Amado RG (2007). Open-label phase III trial of panitumumab plus best supportive care compared with best supportive care alone in patients with chemotherapy-refractory metastatic colorectal cancer. J Clin Oncol.

[B4] Saltz LB, Meropol NJ, Loehrer PJ, Needle MN, Kopit J, Mayer RJ (2004). Phase II Trial of Cetuximab in Patients With Refractory Colorectal Cancer That Expresses the Epidermal Growth Factor Receptor. J Clin Oncol.

[B5] Lenz HJ, Van Cutsem E, Khambata-Ford S, Mayer RJ, Gold P, Stella P, Mirtsching B, Cohn AL, Pippas AW, Azarnia N, Tsuchihashi Z, Mauro DJ, Rowinsky EK (2006). Multicenter Phase II and Translational Study of Cetuximab in Metastatic Colorectal Carcinoma Refractory to Irinotecan, Oxaliplatin, and Fluoropyrimidines. J Clin Oncol.

[B6] Schrag D (2004). The price tag on progress--chemotherapy for colorectal cancer. N Engl J Med.

[B7] Lynch TJ, Bell DW, Sordella R, Gurubhagavatula S, Okimoto RA, Brannigan BW, Harris PL, Haserlat SM, Supko JG, Haluska FG, Louis DN, Christiani DC, Settleman J, Haber DA (2004). Activating mutations in the epidermal growth factor receptor underlying responsiveness of non-small-cell lung cancer to gefitinib. N Engl J Med.

[B8] Paez JG, Janne PA, Lee JC, Tracy S, Greulich H, Gabriel S, Herman P, Kaye FJ, Lindeman N, Boggon TJ, Naoki K, Sasaki H, Fujii Y, Eck MJ, Sellers WR, Johnson BE, Meyerson M (2004). EGFR mutations in lung cancer: correlation with clinical response to gefitinib therapy. Science.

[B9] Pao W, Miller V, Zakowski M, Doherty J, Politi K, Sarkaria I, Singh B, Heelan R, Rusch V, Fulton L, Mardis E, Kupfer D, Wilson R, Kris M, Varmus H (2004). EGF receptor gene mutations are common in lung cancers from "never smokers" and are associated with sensitivity of tumors to gefitinib and erlotinib. Proc Natl Acad Sci U S A.

[B10] Barber TD, Vogelstein B, Kinzler KW, Velculescu VE (2004). Somatic mutations of EGFR in colorectal cancers and glioblastomas. N Engl J Med.

[B11] Chung KY, Shia J, Kemeny NE, Shah M, Schwartz GK, Tse A, Hamilton A, Pan D, Schrag D, Schwartz L, Klimstra DS, Fridman D, Kelsen DP, Saltz LB (2005). Cetuximab Shows Activity in Colorectal Cancer Patients With Tumors That Do Not Express the Epidermal Growth Factor Receptor by Immunohistochemistry. J Clin Oncol.

[B12] Moroni M, Veronese S, Benvenuti S, Marrapese G, Sartore-Bianchi A, Di Nicolantonio F, Gambacorta M, Siena S, Bardelli A (2005). Gene copy number for epidermal growth factor receptor (EGFR) and clinical response to antiEGFR treatment in colorectal cancer: a cohort study. Lancet Oncol.

[B13] Lievre A, Bachet JB, Le Corre D, Boige V, Landi B, Emile JF, Cote JF, Tomasic G, Penna C, Ducreux M, Rougier P, Penault-Llorca F, Laurent-Puig P (2006). KRAS mutation status is predictive of response to cetuximab therapy in colorectal cancer. Cancer Res.

[B14] Di Fiore F, Blanchard F, Charbonnier F, Le Pessot F, Lamy A, Galais MP, Bastit L, Killian A, Sesboue R, Tuech JJ, Queuniet AM, Paillot B, Sabourin JC, Michot F, Michel P, Frebourg T (2007). Clinical relevance of KRAS mutation detection in metastatic colorectal cancer treated by Cetuximab plus chemotherapy. Br J Cancer.

[B15] Khambata-Ford S, Garrett CR, Meropol NJ, Basik M, Harbison CT, Wu S, Wong TW, Huang X, Takimoto CH, Godwin AK, Tan BR, Krishnamurthi SS, Burris HA, Poplin EA, Hidalgo M, Baselga J, Clark EA, Mauro DJ (2007). Expression of Epiregulin and Amphiregulin and K-ras Mutation Status Predict Disease Control in Metastatic Colorectal Cancer Patients Treated With Cetuximab. J Clin Oncol.

[B16] Benvenuti S, Sartore-Bianchi A, Di Nicolantonio F, Zanon C, Moroni M, Veronese S, Siena S, Bardelli A (2007). Oncogenic activation of the RAS/RAF signaling pathway impairs the response of metastatic colorectal cancers to anti-epidermal growth factor receptor antibody therapies. Cancer Res.

[B17] Frattini M, Saletti P, Romagnani E, Martin V, Molinari F, Ghisletta M, Camponovo A, Etienne LL, Cavalli F, Mazzucchelli L (2007). PTEN loss of expression predicts cetuximab efficacy in metastatic colorectal cancer patients. Br J Cancer.

[B18] (1979). WHO handbook for reporting of cancer treatment.. Pathologie Biologie.

[B19] Chung CH, Ely K, McGavran L, Varella-Garcia M, Parker J, Parker N, Jarrett C, Carter J, Murphy BA, Netterville J, Burkey BB, Sinard R, Cmelak A, Levy S, Yarbrough WG, Slebos RJ, Hirsch FR (2006). Increased epidermal growth factor receptor gene copy number is associated with poor prognosis in head and neck squamous cell carcinomas. J Clin Oncol.

[B20] Sartore-Bianchi A, Moroni M, Veronese S, Carnaghi C, Bajetta E, Luppi G, Sobrero A, Barone C, Cascinu S, Colucci G, Cortesi E, Nichelatti M, Gambacorta M, Siena S (2007). Epidermal Growth Factor Receptor Gene Copy Number and Clinical Outcome of Metastatic Colorectal Cancer Treated With Panitumumab. J Clin Oncol.

[B21] Shia J, Klimstra DS, Li AR, Qin J, Saltz L, Teruya-Feldstein J, Akram M, Chung KY, Yao D, Paty PB, Gerald W, Chen B (2005). Epidermal growth factor receptor expression and gene amplification in colorectal carcinoma: an immunohistochemical and chromogenic in situ hybridization study. Mod Pathol.

[B22] Sauer T, Guren MG, Noren T, Dueland S (2005). Demonstration of EGFR gene copy loss in colorectal carcinomas by fluorescence in situ hybridization (FISH): a surrogate marker for sensitivity to specific anti-EGFR therapy?. Histopathology.

[B23] Al-Kuraya K, Novotny H, Bavi PP, Siraj AK, Uddin S, Ezzat A, Al Sanea N, Al-Dayel F, Al-Mana H, Sheikh SS, Mirlacher M, Tapia C, Simon R, Sauter G, Terracciano L, Tornillo L (2006). HER2, TOP2A, CCND1, EGFR, And C-MYC oncogene amplification in colorectal cancer. J Clin Pathol.

[B24] Li S, Schmitz KR, Jeffrey PD, Wiltzius JJW, Kussie P, Ferguson KM (2005). Structural basis for inhibition of the epidermal growth factor receptor by cetuximab. Cancer Cell.

[B25] Moriai T, Kobrin MS, Korc M (1993). Cloning of a variant epidermal growth factor receptor. Biochem Biophys Res Commun.

[B26] Wang WS, Chen PM, Chiou TJ, Liu JH, Lin JK, Lin TC, Wang HS, Su Y (2007). Epidermal growth factor receptor R497K polymorphism is a favorable prognostic factor for patients with colorectal carcinoma. Clin Cancer Res.

[B27] Lassman AB, Rossi MR, Raizer JJ, Abrey LE, Lieberman FS, Grefe CN, Lamborn K, Pao W, Shih AH, Kuhn JG, Wilson R, Nowak NJ, Cowell JK, DeAngelis LM, Wen P, Gilbert MR, Chang S, Yung WA, Prados M, Holland EC (2005). Molecular study of malignant gliomas treated with epidermal growth factor receptor inhibitors: tissue analysis from North American Brain Tumor Consortium Trials 01-03 and 00-01. Clin Cancer Res.

[B28] Zhang W, Stoehlmacher J, Park DJ, Yang D, Borchard E, Gil J, Tsao-Wei DD, Yun J, Gordon M, Press OA, Rhodes K, Groshen S, Lenz HJ (2005). Gene polymorphisms of epidermal growth factor receptor and its downstream effector, interleukin-8, predict oxaliplatin efficacy in patients with advanced colorectal cancer. Clin Colorectal Cancer.

[B29] Moriai T, Kobrin MS, Hope C, Speck L, Korc M (1994). A Variant Epidermal Growth Factor Receptor Exhibits Altered Type {alpha} Transforming Growth Factor Binding and Transmembrane Signaling. PNAS.

